# A dietary intervention study to reduce Metabolic Syndrome risks in heart-transplanted patients

**DOI:** 10.1093/eurpub/ckac131.240

**Published:** 2022-10-25

**Authors:** G Valdi, V Ferrara, M Marinoni, C Nalli, C Di Nora, S Sponga, G Benedetti, M Parpinel, U Livi, V Moretti

**Affiliations:** 1 DAME, Università di Udine, Udine, Italy; 2 Cardiothoracic Department, Friuli Centrale Healthcare and University Trust, Udine, Italy

## Abstract

**Background:**

Since heart transplantation (HTx) has become the gold standard therapy in end-staged heart failure, many factors, including metabolic syndrome (MS), represent a burden in HTx patients. Considering key role of immunosuppressive therapy and its side effects on the appearance of MS, we focused on modifiable factors including adherence to Mediterranean Diet (MD) and improvement of dietary habits.

**Methods:**

21 heart-transplanted patients were enrolled and randomized in a control group (CG; N 10) and an intervention group (IG; N 11). During two meetings (baseline, 6-month follow-up) were administered a validated Food Frequency Questionnaire (FFQ), to assess adherence to MD, and collected clinical and anthropometric parameters, IG were additionally requested to fill a food diary. IG received personalized advices, CG received standard recommendations. Comparison between IG and CG were analyzed, differences into the IG were also assessed.

**Results:**

The prevalence of MS at baseline was 46% in IG and 20% in CG. During 6-month follow-up, significant lower blood pressure values were observed (median, 25th-75th: systolic 130, 120-130 IG vs 145, 130-147 CG; p = 0.004). Seven patients of IG underwent a 12-month meeting. In this group MD scores increased significantly (7 + 1.3 vs 4 + 1.5, p = 0.001). Furthermore, significant decrease of fat mass percentage (%) (23.3 + 6.3 vs 14.8 + 10.1, p = 0.014), increase of fat free mass % (76 + 6.3 vs 85.2 + 10.1, p = 0.014) and increase of body cell mass % (50.9 + 3.8 vs 53.4 + 3.4, p = 0.031) were observed. Dietary data in IG showed significant decrease of energy from saturated fatty acids % (13.0±2.1 vs 9.6±1.5, p = 0.001), sodium (mg) (2138±359 vs 1822±417, p = 0.045), and decreasing trend for cholesterol (mg) (219±82 vs 171±59, p = 0.082).

**Conclusions:**

Dietary intervention based on MD perhaps can improve MS risks in heart-transplanted patients. Further investigations may be needed to assess the fundamental role of a structured nutritional follow-up in these patients.

**Key messages:**

• Personalized nutritional advices based on the MD, compared to general recommendation, can significantly improve health and quality of life in heart-transplanted patients.

• A structured nutritional follow-up for heart-transplanted patients may be desirable to prevent risks of Metabolic Syndrome as a public health instrument in selected categories as these patients.

